# SMA micro-hand implemented in small robot for generating gestures

**DOI:** 10.1007/s11370-021-00364-9

**Published:** 2021-04-22

**Authors:** Ishikawa Takumi, Nagasawa Sumito

**Affiliations:** 1 Department of Mechanical Engineering, Graduate School of Science and Engineering, Shibaura Institute of Technology, 3-7-5 Toyosu, Koto-ku, Tokyo, 135-8548 Japan; 2 Department of Engineering Science and Mechanics, Shibaura Institute of Technology, 3-7-5 Toyosu, Koto-ku, Tokyo, 135-8548 Japan

**Keywords:** Shape memory alloy, Robot hand, Micro-robot, Gesture

## Abstract

Research on robots that can be used for communication with humans has become popular in recent years. Communication robots should ideally be as small as an infant in order to reduce the user’s feeling of threat. In addition, non-verbal communication (such as gestures) is also important in facilitating smooth interactions between humans and robots. There are currently a few communication robots that are small sized and can generate hand gestures. In this paper, we propose a small robot hand, which is optimized for gesture communication by using a shape memory alloy (SMA). The SMA employed is a Ti–Ni alloy, which is used as an actuator. The SMA shrinks when it transforms into the austenite phase at temperatures higher than the transformation temperature. When it is in the martensitic phase at a lower temperature, it is expanded by an external force. Each finger of the robot hand is driven by an individual SMA wire. The specifications of the small robot including the hand size, operation angles in each finger joint, response times and power consumption were determined according to the human finger and existing small communication robots. These required specifications have been fulfilled by carefully designing the geometry and heating/cooling power control. A questionnaire-based survey was also conducted with a robot hand. The five-finger hand was successfully shown to generate recognizable symbolic gestures.

**Supplementary Information:**

The online version supplementary material available at 10.1007/s11370-021-00364-9.

## Introduction

### Background

In recent years, research on robots for communication with humans has become very active [1–3]. The main purpose of these robots is to serve and live with them as human partners, assistants, or companions. Service robots are beginning to become part of their jobs in many areas, including shopping, education and companions. When deploying robots in public and private locations, robots need to be able to provide natural services. Manipulating appearances, facial expressions, voices and gestures can improve the service quality of robots and improve their social acceptability [4]. Previous researchers suggested that humans could communicate easily with robots that have physical features similar to humans, such as arms and a head [5, 6]. Most robots on the market today, such as Pepper, Nao and PALRO, tend to have a humanoid appearance. In addition, various studies have shown that humanoid robots are more likely to be accepted by people than other types of robots. Additionally, the size of communication robots for home use should not be too large, and ideally should be as small as an infant [7, 8].

In human communication, verbal information and non-verbal information are generally used. Non-verbal communication includes all communication except speech (facial expressions, gestures, gaze, etc.). In particular, non-verbal information accounts for 60–70% of the total amount of information exchanged among humans [9, 10]. By using non-verbal information, humans can additionally communicate cognitive information such as information about the surroundings, their feelings, emotions, etc. Cognitive information adds a lot of meaning to the logical information communicated verbally. Non-verbal communication is powerful in communicating the emotions and attitudes of the speaker and complementing linguistic communication [11, 12]. This might be the same in the case of communications between humans and robots. Earlier studies have reported that communication between humans and robots becomes better using gesturing robots than using the ones that do not use gestures [13–15].

**Fig. 1:**
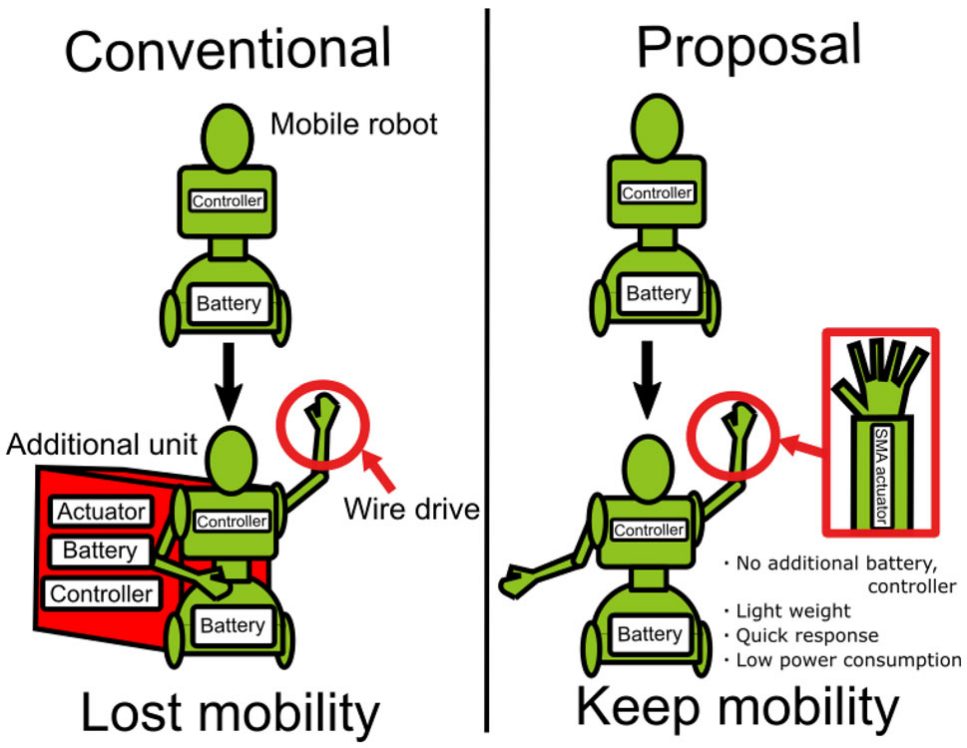
Proposal small robot hand

There are many previous research studies on robot hands; however, the robots considered in these studies have hands almost the size of human hands, and the hands are only meant for grasping objects. There are few studies concerning small robot hands having operational fingers. One of the smallest developed robot hands is used in laparoscopic surgery [16]. However, the driving mechanism, the controller and the base of the power source for such robot hands are too large to be mounted on a small robot (Fig. 1). Therefore, if an existing five-finger small robot hand is mounted on a robot, the large base unit will impair its mobility and appearance. The new proposal of this paper is to develop a gesture robot hand that fits in the forearm of a small robot, and to maintain mobility and give the small robot a finger drive for gestures. Another small robot hand, the SMA robot hand that is 1/3 the size of a human, has been reported [17]. This robot hand has 20 degrees of freedom for gripping small parts. The power consumption of the SMA robot hand is not evaluated in this paper. It is necessary to quantitatively evaluate the power consumption for the purpose of mounting on a small robot. Hands with five fingers can express considerable amount of information [18, 19]. For instance, symbolic motions that are substituted for specific words like the V-sign, or instructional motions that express something, such as using the index finger for pointing directions. Many researchers are trying to classify the various gestures that accompany utterances. Nehaniv et al. classified five gesture types in order to infer the intent of gesture. These five classes are irrelevant gestures, side effect of expressive behavior, symbolic gestures, interactional gestures and referential gestures [20]. Finger gestures are mainly included in symbolic gestures. According to Nehaniv et al, examples of symbolic gestures are peace signs, salutes and more. Therefore, small robot hands with five fingers are necessary for the robots to be able to communicate effectively with the users. The purpose of this study is the design, fabrication and demonstration of a small robot capable of using hand gestures.

### Requirement

**Fig. 2:**
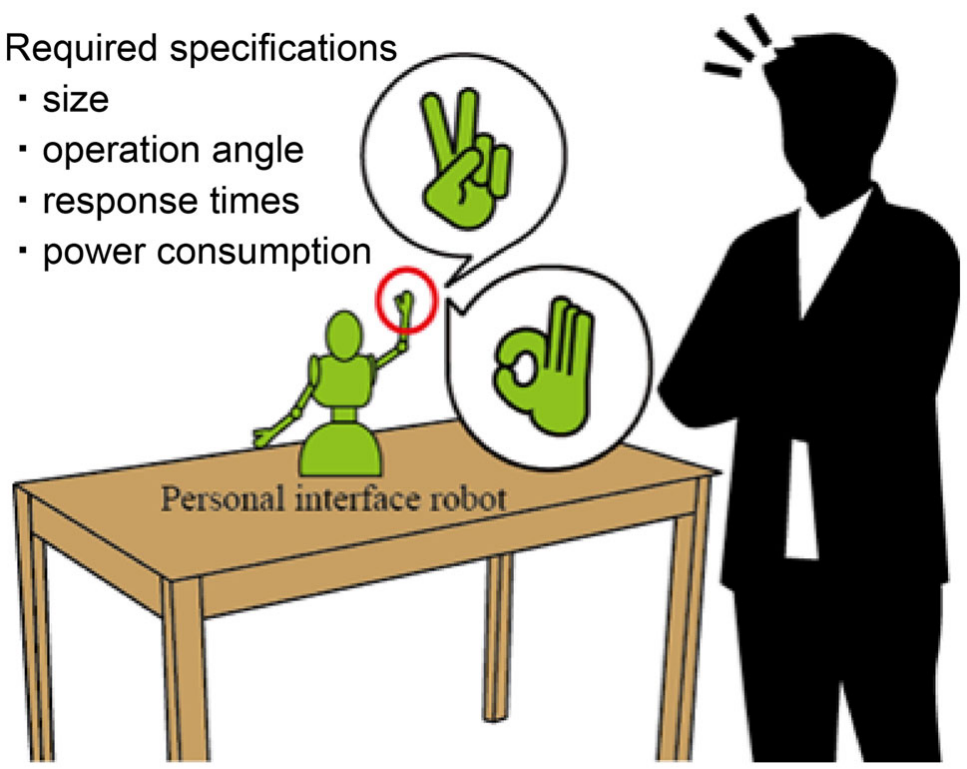
Gesture communication by a small robot

As shown in Fig. 2, there are four requirements for the design of our robot hand. The first is that the robot hand size, comprising the driving mechanism, controller and the power source, must be small enough to be attached to a small robot. Secondly, the operational angles of the finger joints should be wide enough for a variety of gestures. Thirdly, it should evoke responses in a short time to preempt giving unnatural impression to the user. The last is that the power consumption has to be small enough to be implemented in a small robot. Our robot hand is assumed to be implemented in a small robot whose height is about $H_r=400$ mm. The length from the tip of the middle finger to the wrist $L_r$ is determined by referring to the human body dimension data $L_h$ and the ratio of the robot height to human height. The representative dimension of the robot hand is calculated using the following equation:

1 $$\begin{aligned} L_r=L_h\cdot \frac{H_r}{H_h} \end{aligned}$$

$L_r$ is the representative dimension of the robot hand, $L_h$ the length from human fingertip to wrist, $H_r$ the height of the communication robot, and $H_h$ the average height of a Japanese.

$H_h=1654.7$ mm, $H_r=400$ mm, $L_h=182.6$ mm [21], then, $L_r$ is 45 mm, as calculated from Eq. (1). In this study, our robot hand is designed to satisfy the dimension $L_r$.

**Fig. 3:**
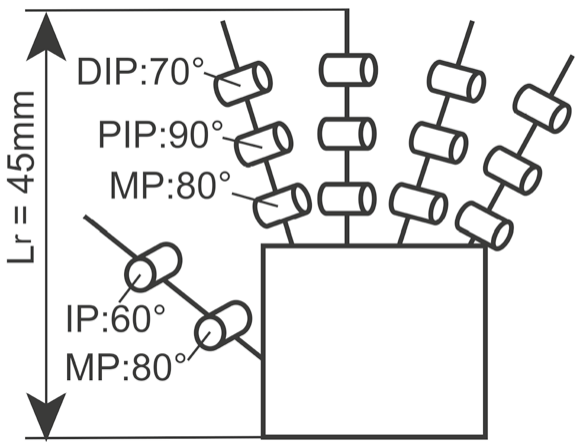
Requirement specifications for our robot hand

For gesture expression, our fingers are appropriate to operate about 90 % of the ranges of the human finger joints. Referring to the range of human finger joints, the following operational angles are required: the metacarpo-phalangeal (MP) joint angle $\theta _\mathrm{MP}=80\,^\circ$, the proximal inter-phalangeal (PIP) joint angle $\theta _\mathrm{PIP}=90\,^\circ$ and the distal inter-phalangeal (DIP) joint angle $\theta _\mathrm{DIP}=70\,^\circ$ (Fig. 3).

The system reaction time limit, generally allowed by the user, is known to be 2 s [22–24]. This is widely used in the design of various human interfaces such as the GUI (Graphical User Interface), television and computers. For this reason, the required response time of the finger of our robot hand is set to be less than 2 s, during the extending and the bending of the fingers.

The required power consumption was determined considering the specifications of the small communication robot PALRO from FUJISOFT INCORPORATED [25]. When PALRO is walking, the power consumption is at its maximum of 60 W, and during conversations, the power consumption is 30 W. So, there is a 30 W margin for driving our fingers. Therefore, the required specification for the power consumption of our hand is less than 15 W for each hand.

The required specifications of the drive angle, response time and power consumption are evaluated by two experiments. The drive angle and response time are measured by image analysis, by attaching markers at the tip of the robot’s finger and at each joint. The power consumption is evaluated by measuring the drive current and the voltage of our robot hand.

## Method

### Ti–Ni alloy

Shape memory alloy (SMA) was used in the actuators of our robot hand. Even if the SMA is deformed into an arbitrary shape, it will recover to its original shape when heated above its own temperature. Large strain remains in SMA in the low temperature range of $T\le 298$ K, but the strain disappears when heated above the Austenite phase transformation end temperature. The shape memory effect appears in this temperature range. This shape memory effect is caused by a phase transformation from the austenite phase to the martensite phase or from the martensite phase to the matrix phase. NiTi alloys and copper-based and some iron-based alloys have been put into practical use. NiTi alloy is excellent in shape recovery characteristics and repeatability. It is effective for SMA to use both the temperature sensing function and the actuator function at the same time. The practical application of SMA has progressed in this way, and it will be applied as a sensor and actuator to home appliances, automobiles and trains. The SMA has been used for small robots since the early 1990s [26–28]. The first important feature of the SMA is miniaturization. The ratio of output power to the weight of the SMA is quite high. Therefore, the SMA is suitable for miniaturization and in the actuators of small robot hands. The second important feature is smooth movement, as the movement of the SMA is similar to that of a living thing. Therefore, it is considered suitable for building robot legs and arms actuators that move flexibly. Biometal fiber (Toki Corporation, BMF) is a wire-like SMA that contracts and stretches like a muscle. When its temperature is below the transition temperature, it is soft like a nylon thread. However, it becomes hard when a current is applied, and its temperature rises above the transformation temperature, at which it shrinks with a strong force. It softens again when the current is stopped. The operating life of the BMF is long, and the BMF100 which a type of biometal fiber, has a diameter of 100 m, can be moved a 100 million times or more under a load stress of 100 MPa. Furthermore, BMF has a better response than the other SMA actuators, and the BMF100 can react at 3 Hz in a windless room temperature ($20\,^\circ$C) [29]. Table 1 shows the main specifications of BMF100 [30]. In this study, BMF100 with a diameter of 0.1 mm was used as an actuator.

**Table 1:** Specification of BMFs from Toki Corporation [30]

	BMF50	BMF75	BMF100	BMF150
Standard diameter (mm)	0.05	0.075	0.1	0.15
Practical force produced (gf)	18	35	70	150
Kinetic displacement (%)	4.0	4.0	4.0	4.0
Standard drive current (mA)	80	140	200	340
Standard drive voltage (V/m)	42.2	35.4	27.0	20.7
Standard power (W)	3.37	4.63	5.40	7.05
Standard resistance ($\Omega$/m)	528	238	135	61

### Design of the SMA robot hand

The robot hand was designed for gestures, so one aim was for it to make gestures such as V-signs. Anatomically, a human hand has about 20 degrees of freedom. Owing to the difficulties involved in formulating a finger of the desired small size with multiple degrees of freedom, each finger was designed to only bend and extend. Each finger, excluding the thumb had four links, i.e., equivalent to the middle phalanx, the proximal phalanx, the distal phalanx and the body of the phalanges. Each joint, i.e., the MP joint, PIP joint and DIP joint has one degree of freedom of bending.

**Fig. 4:**
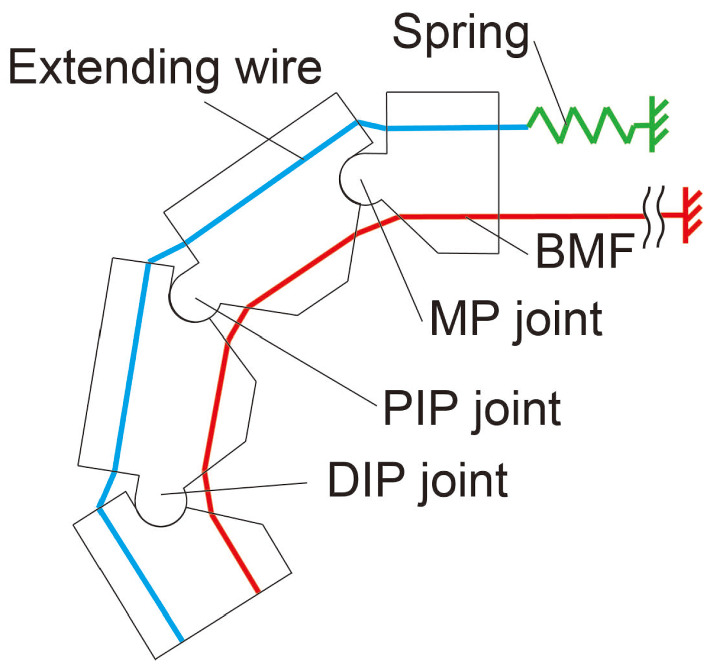
Structure of a robot finger

**Fig. 5:**
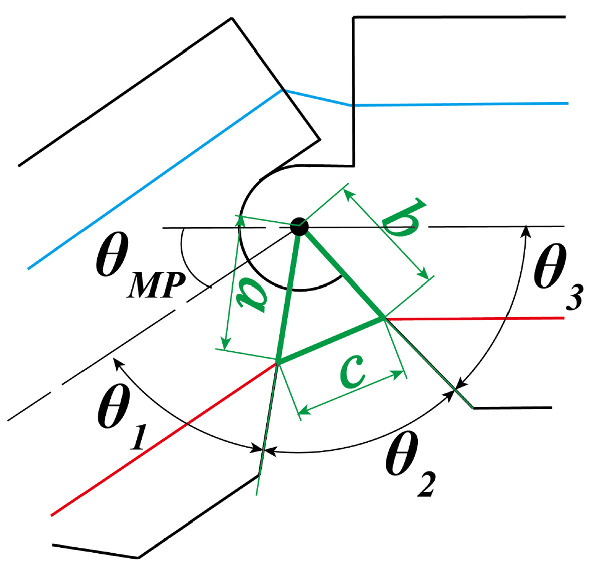
The joint of a robot finger

In order to achieve gestures such as the V-sign or thumbs-up, the bending order of the three joints is not important. Our robot finger was designed in such a way that about the three joints were bent by one BMF. To obtain enough SMA deformation for bending the robot finger sufficiently using one BMF, it is necessary for the wire length to be longer than the robot hand. Therefore, the BMF is mounted at a point near the elbow in the forearm from which it is possible to achieve the requirements of the drive angles of the finger joints. The bending/extending mechanism of the robot hand was based on the human tendon drive structure [31]. The BMF for bending was placed on the palm side of the finger, and the bias spring and the connecting wire for extending were placed on the dorsal side. As shown in Fig. 4, each hinge of the joint was formed by a convex and a concave that were arc-shaped and joined. The hinges were held with the tension of the extending wire and the BMF passing through each phalanx. Due to this mechanism, the hinges were constructed without any shafts, and this mechanism was also helpful in reducing the size of the finger. The drive mechanism was designed with a one-way active bending mechanism [32]. A large bending angle can be obtained even with a small amount of displacement. The amount of BMF displacement depends on the positions of the holes (shown in Fig. 5, the length of a and b), the BMF passes the holes(shown in Fig. 5 by red line). The bending angle increases as the distance between the point of action of the generated force by the SMA and the central axis of rotation of the finger becomes closer. Figure 5 shows the MP joint. The operation range of the MP joint $\theta _\mathrm{MP}^\mathrm{max}$ was designed to be up to $80\,^\circ$, according to the required specifications. In order to design the operation range of the MP joint, the angle $\theta _2$ shown in Fig. 5 is determined in Eq. (2) as follows:

2 $$\begin{aligned} \theta _2 (t)=180-\theta _MP (t)-\theta _1-\theta _3. \end{aligned}$$

$\theta _1$ and $\theta _3$ were designed as: $\theta _1=45\,^\circ$ and $\theta _3=55\,^\circ$, so that $\theta _2$ can change between 0 and $80\,^\circ$, which corresponds to $\theta _\mathrm{MP}^\mathrm{max}$. Therefore, when $\theta _\mathrm{MP}$ is 80, the distance *c* is zero and when $\theta _\mathrm{MP}$ is zero, the distance *c* is the BMF displacement required for the bending of the MP joint to $\theta _\mathrm{MP}^{max}=80\,^\circ$. The distance *c* is determined by Eq. (3).

3 $$\begin{aligned} c=\sqrt{a^2+b^2-2abcos \theta _2} \ \ |\ (\theta _\mathrm{MP}=0) \end{aligned}$$

Similarly, the distances c at the MP, PIP and DIP joints were calculated as $c_\mathrm{MP}$, $c_\mathrm{PIP}$, $c_\mathrm{DIP}$. The BMF displacement $\delta$ is the sum of these distances as shown:

4 $$\begin{aligned} \delta =c_\mathrm{MP}+c_\mathrm{PIP}+c_\mathrm{DIP}. \end{aligned}$$

The displacement of BMF $\delta$ was calculated as 10.00 mm by Eq. (4). The total length of the BMF was calculated as $L = 500$ mm from Eq. (5), because the strain of the BMF $\epsilon = 4\%$. Both the ends of the BMF are mounted at a point near the elbow in the forearm and turned back at the tip of the finger. Therefore, the length of the BMF is twice the length from the tip of the finger to the mounted point.

5 $$\begin{aligned} L=2\cdot \frac{100}{\epsilon }\cdot \delta . \end{aligned}$$

The specifications of the bias spring were calculated from Eq. (6). The spring tension has to be smaller than the generated force of the BMF shown in Table 1 For designing the spring tension, a significant consideration is needed, because when the tension is too weak, the response of the finger during extending becomes slow, and when its tension is too strong the response of the finger during bending becomes slow. Both responses, extending and bending, have to take less than 2 s. Both the ends of the BMF are mounted at a point near the elbow in the forearm and turned back at the tip of the finger. Therefore, the force of the BMF is twice the generated force of the BMF in Table 1. The force to bend the finger by BMF is calculated as 1.372 N. $P_i=0.265$ N, $k=0.034$ N mm, $L_i=43$ mm, $L_0=21$ mm, then, $T_\mathrm{spr}$ is 1.353 N, as calculated from Eq. (6).

6 $$\begin{aligned} T_\mathrm{spr}=P_i+k(L_i-L_0+\delta ) \end{aligned}$$

$T_\mathrm{spr}$ is the spring tension, $P_i$ the initial tension, *k* the spring constant, $L_0$ the initial length $L_i$ the installation length, and $\delta$ the displacement of BMF.

**Fig. 6:**
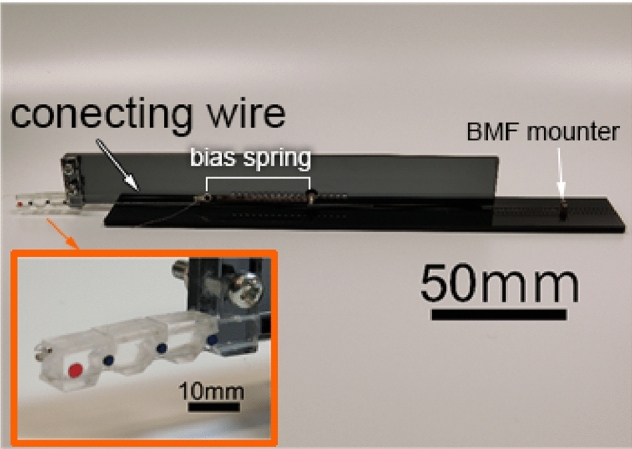
SMA robot finger

**Fig. 7:**
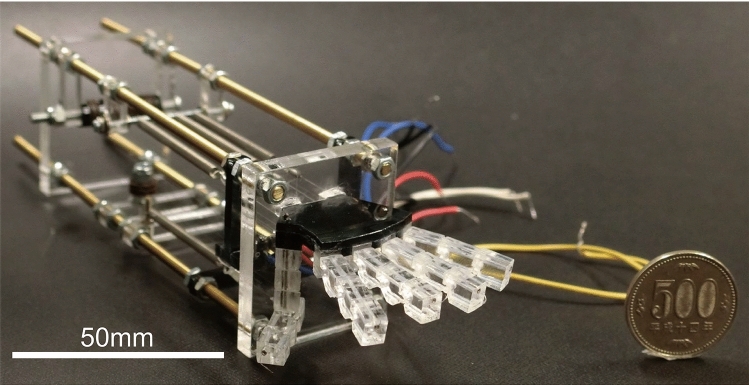
SMA robot hand

A finger prototype of the robot hand was fabricated. Figure 6 shows the prototype. The fingers were made by cutting acrylic resin with a $\text {CO}_2$ laser cutter. The operating angles and responses of the joints of this finger were measured by video analysis. A five-fingered robot hand was then fabricated and its power consumption was evaluated. Figure 7 shows the five-fingered robot hand. The dimensions of the robot hand from the tip of the finger to the wrist were $L_r= 44$ mm, and the overall dimensions were 161 mm $\times$ 46 mm $\times$ 40 mm. The thumb was made from two joints and its driving direction was perpendicular to those of the other fingers.

### Experiments of operation angle and response

**Fig. 8:**
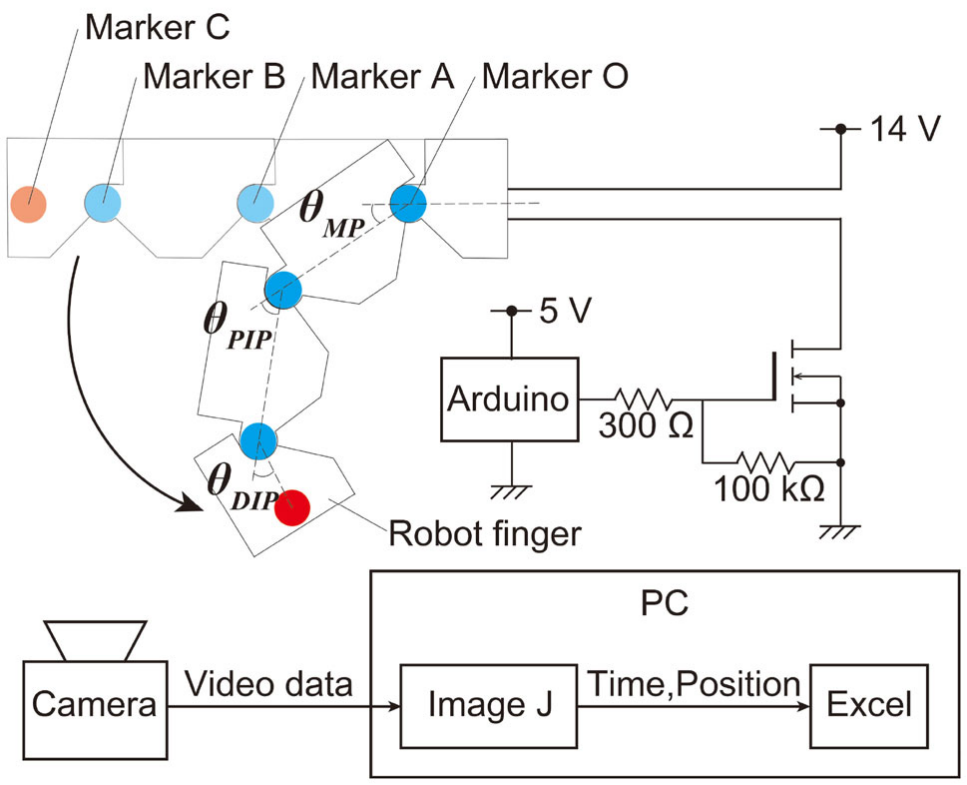
Evaluation of the operation angle and response

The video analysis using the ImageJ software was used to measure the response time and operation angles of the robot finger. The basic form of a measurement of angles and response time is described in Fig. 8, and this experiment was performed to check whether the target requirements were satisfied. This experimental setup comprised a robot finger, a control circuit, an MCU, a power supply, a video camera and a processing PC. Markers were attached to the tips and each joint of the robot finger, and the relationship between time and the current joint angle was measured by image analysis using the video. The power feed of the SMA was changed by pulse width modulation (PWM). The BMF was energized for 15 s with 14 V, and the fingers were bent; then, the BMF was air-cooled for each extending joint, and a video was taken at 30 fps. By observing the LED changes from turning on to off in the video analysis, we can determine the switch timing from SMA heating to cooling. The LED is on when the SMA is heating and off when it is cooling. The LED flicker is not a problem because the LED blink frequency is 100 Hz and the video frame rate is 30fps. The ratio of the turn-on duration to the period used in the PWM control was defined as the duty ratio *D*[%]. The SMA actuators are often driven by a pulse width modulation (PWM) driver [33]. The input of the SMA wire is a duty ratio. The SMA wire is in the martensite state when the actuator is not driven with the duty ratio of zero. Assume that a temperature reaches at the steady state for a duty ratio of one. If is much higher than, overheating occurs and causes serious damage to the memorized shape. Moreover, higher values of increase the heating rate, but do not increase cooling rate. In this measurement, the SMA heating with $D = 100\%$ lasts for the first 3 s; then, the heating with a minimum duty ratio $D = 45\%$, which can keep the robot finger bending, lasts for the subsequent 12 s. The positions of the markers $O(x_o,y_o) A(x_a,y_a) B(x_b,y_b)$ and $C(x_c,y_c)$ in Fig. 8 were measured using the image analysis software (ImageJ). The angles $\theta _\mathrm{MP}$, $\theta _\mathrm{PIP}$ and $\theta _\mathrm{DIP}$ of each joint were calculated from the marker positions by Eqs. (7–9).

7 $$\begin{aligned}&\theta _\mathrm{MP}=\tan ^{-1}\left( \frac{y_o-y_a}{x_o-x_a}\right) \end{aligned}$$

8 $$\begin{aligned}&\theta _\mathrm{PIP}=180-\cos ^{-1}\left( \frac{|OA|^2+|AB|^2-|OB|^2}{(2\cdot |OA|\cdot |AB|}\right) \end{aligned}$$

9 $$\begin{aligned}&\theta _\mathrm{DIP}=180-\cos ^{-1}\left( \frac{(|BC|^2+|AB|^2-|AC|^2}{((2\cdot |BC|\cdot |AB|}\right) . \end{aligned}$$

Regarding the response time, it was difficult to determine the finishing time of the finger with complete bending/extending; this is because there is a slight fluctuation in the angle data, and the response becomes slow, when the BMF temperate is close to the room temperature ($20\,^{\circ }$C) . Therefore, the rising and the falling times were used as the evaluation indexes for the robot finger bending and extending. The time, from the start of the heating/cooling to the time when all three joints were bent/extended to 90% of the required operation angle, was defined as the bending time/the extending time. These were compared with the required specification of 2 s.

**Fig. 9:**
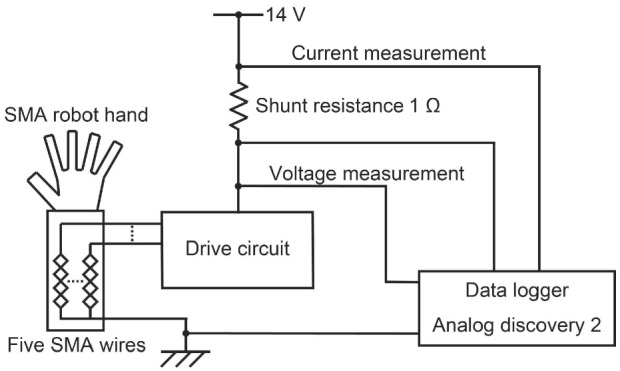
Evaluation of the power consumption

### Experiments on power consumption

The basic form of an evaluation of the power consumption is described in Fig. 9. The objective is to evaluate whether the power consumption satisfies the requirement of 15 W. The experimental setup comprised a robot hand, a shunt resistance of 1 $\Omega$, the data logger Analog discovery 2 from the Digilent, Inc., and a stabilized DC power supply.

The power consumption is the maximum at the moment when the five fingers are simultaneously activated. The time variation of the circuit voltage and current was measured at the time the five fingers of the robot hand were driven. The measurement was also performed by heating, with $D = 100\%$, during the first 3 s and with the minimum duty ratio $D = 40\%$ that could sustain the bending of the robot finger for subsequent 15 s. However, the commencement of the bending of the thumb was delayed by 0.5 s to avoid interference with the other fingers. The power consumption was calculated from the measured voltage and current.

### Questionnaire survey of gesture

A questionnaire survey using a robot hand was also conducted. The objective is to evaluate whether the generated gesture is recognized and the smoothness of the gesture. Four questions were developed and distributed to 15 students in the laboratory. Considering the 3 essentials of prevention in the COVID-19 pandemic, respondents answered the questionnaire by watching the video of gesture generation. Three types of videos are prepared for each type of gesture.

In the first video, the thumb, ring finger and little finger are bent to generate a peace sign (Video 1). The peace sign was selected because it is a typical gesture that is classified as Symbolic Gestures or emblems. In the second video, the thumb and index finger are bent and their fingertips touch each other to generate an OK sign (Video 2). The OK sign was chosen because it seems more difficult to generate and perceive than the peace sign. In the third video, the index finger, middle finger, ring finger and little finger repeatedly bend and extend to generate a “Come on” gesture (Video 3). It was selected because it is a dynamic gesture. After watching the three videos, the respondents chose from four options: “I understood,” “I could understand somewhat,” “I didn’t understand much” and “I didn’t understand at all.” This item is related to the validity of the driving angle of the robot hand when generating a symbolic gesture. Regarding the drive of gestures, Respondents also chose from four options: “I felt unnatural,” “I felt a little unnatural,” “I didn’t feel unnatural,” and “I didn’t feel unnatural at all.” This item is related to the validity of the responsiveness of the robot hand. If the participants do not feel unnatural in driving, the robot hand can provide a more human-like gesture. In order to know future issues and the feelings of the respondents, a free text box was provided at the end of the questionnaire. The results of the questionnaire survey were aggregated by simple tabulation.

**Fig. 10:**
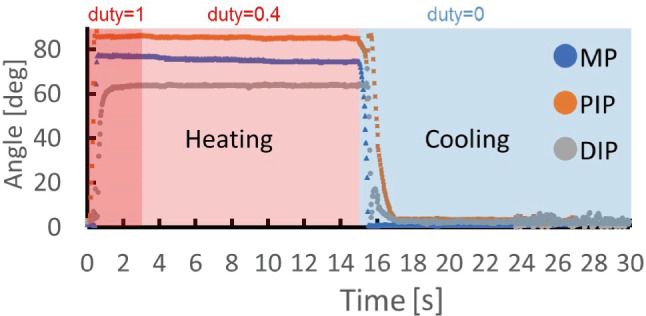
Relations of angle and time

**Fig. 11:**
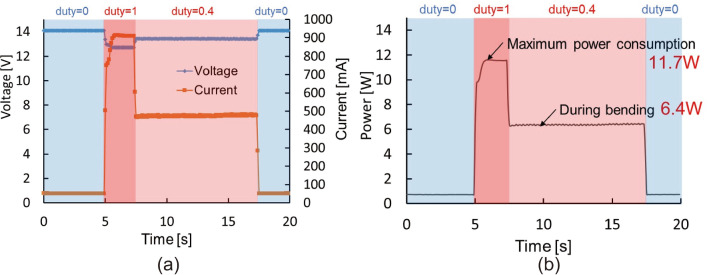
Relations of the power consumption and time

## Results and discussion

### Discussion of angles and responsiveness

Figure 10 shows the relationship between the current joint angle and time, with the horizontal axis representing time and the vertical axis representing the angle. The heating duration is displayed in red, and at the end of the heating duration the supply of power is stopped, and then the cooling duration starts and is displayed in blue. The response of each joint is shown in the MP, PIP and DIP series, respectively.

As a result, the bending angle of each joint reached 91% of the required specification. The remaining 9% is probably due to an error in the position of the hole, through which the BMF passed, or due to the clearance between the BMF and the hole. The response time was 1.87 s for bending and 1.63 s for extending. Even during the use of duty ratio $D = 45\%$, each joint maintained its bending state without starting extension. The bending state was maintained, because the amount of Joule heating and the amount of heat cooling are balanced. At that time, the extending time was 1.63 s, which was within the required specification. The result was that the bending/extending time was within the required specification. If the Joule heating of BMF was stronger than the cooling, the SMA temperature became too high for extension in the required time.

### Discussion of the power consumption

Figure 11a shows the relationship between the SMA voltage/current and time, with the horizontal axis representing time and the vertical axis representing the voltage/current. Figure 11b shows the relationship between the power consumption and time, with the horizontal axis representing time and the vertical axis representing power. The BMF voltage dropped from 14 V to 12.7 V at the start of bending, and it was 13.4 V while maintaining the bend. The voltage dropped due to the internal resistance of the power supply. The maximum current used was 910 mA and it was 480 mA while maintaining the bend. As a result, the maximum power consumption was 11.7 W, which is below the required specification of 15 W. Moreover, it was only 6.4 W while holding the bend.

### Discussion of questionnaire survey

Four questions were developed and distributed to 15 students in the laboratory, with a response rate of 93%. Participants consist of 14 students from laboratory. The sample consists of 3 women (20%) and 12 men (80%).

**Fig. 12:**
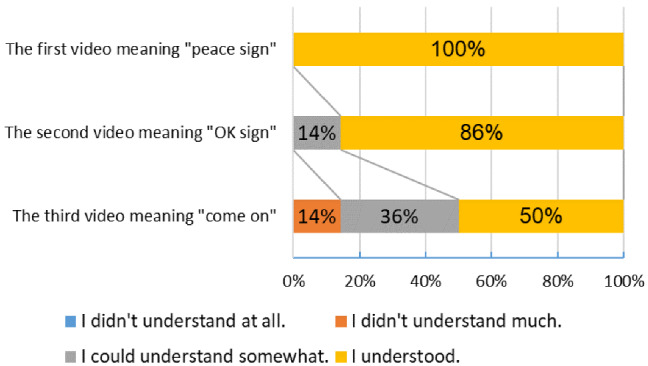
Results of gesture perceive questionnaire

In the first video meaning “peace sign,” 14 of them (100%) indicate that they understood the meaning of gestures clearly (Fig. 12). In the second video meaning “OK sign,” 12 of them (86%) indicate that they understood the meaning of gestures clearly, 2 of them (14%) indicate that they could understand somewhat. The driving angle of the robot’s hand was sufficient to generate static symbolic gestures. Some respondents were difficult to recognize because the ring made by the thumb and index finger was distorted. In the third video meaning “Come on,” 7 of them (50%) indicate that they understood the meaning of gestures clearly, 5 of them (36%) indicate that they could understand somewhat and 2 of them (14%) indicate that they didn’t understand much. The third video assumed a “Come on” gesture, but many respondents recognized it as a thumbs-up, according to the free text box. The cause is that the finger drives are out of sync.

**Fig. 13:**
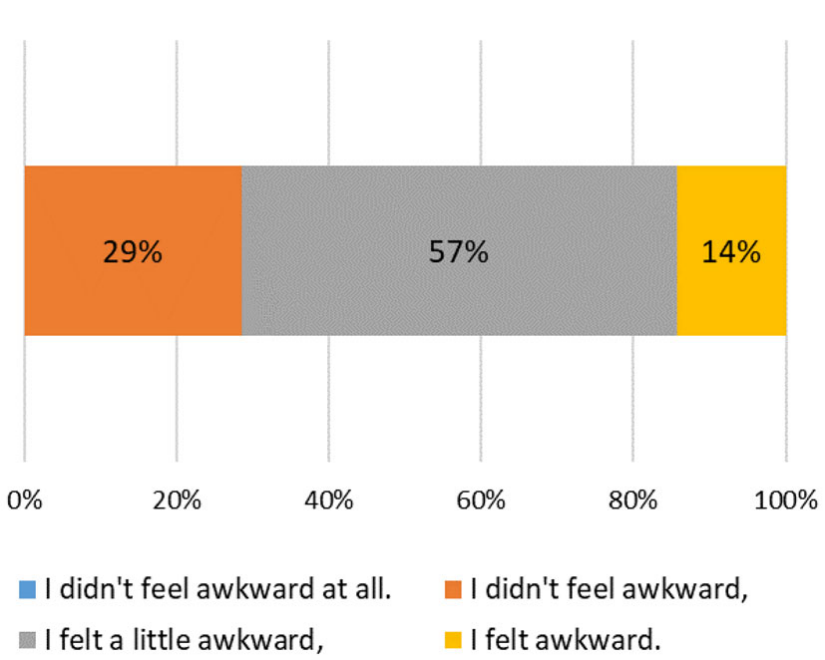
Results of the gesture unnaturalness questionnaire

Regarding the unnaturalness of gestures, 2 of them (14%) indicate that they felt unnatural, 8 of them (57%) indicate that they felt a little unnatural and 4 of them (29%) indicate that they didn’t feel unnatural (Fig. 13). Many respondents find it unnatural when multiple fingers are out of sync. It seems that the reason why the finger drive is not synchronized is that the tensions of the five BMFs are not the same and that the training process of the BMFs is also insufficient [34].

**Fig. 14:**
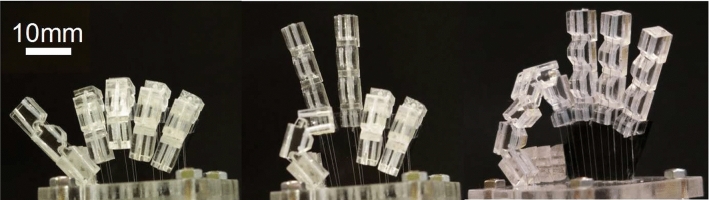
Hand gestures of the SMA robot hand

Figure 14 shows some typical gestures such as a thumbs-up, a peace sign and an OK sign. Since the three joints are driven by the one actuator, each joint cannot bend to an arbitrary angle, but by adjusting the duty ratio, it was possible to maintain a lightly bent state, like that of the index finger with an OK sign.

## Conclusion

The communication between humans and robots becomes smoother by using gestures. Moreover, to alleviate the feeling of threat, a small robot, the size of an infant, is suitable. Therefore, we proposed the development of an SMA robot hand for a small interface robot that communicates with humans using gestures. Many studies using SMA for robot hand actuators are aimed at gripping and are the same size as human hands. On the other hand, the small robot hand with five fingers in the previous research has a large base unit and cannot be attached to a small robot. In this paper, a robot hand with a hand part of 45 mm and an overall length of 161 mm $\times$ 46 mm $\times$ 40 mm has been developed and can be mounted on an existing small robot with a height of 40–50 cm. Four specifications were required for our robot hand. The distance from the wrist to the middle fingertip was set to 45 mm, for mounting on a small robot. In order to express gestures, the robot hand requires a driving angle that bends the MP joint to $\theta _\mathrm{MP}=80\,^\circ$, the PIP joint to $\theta _\mathrm{PIP}=90\,^\circ$, and the DIP joint to $\theta _\mathrm{DIP}=70\,^\circ$. In order to avoid giving the user an unnatural impression, the required response time of the fingers of the robot hand is set to be less than 2 s, for each case of extension and bending. Considering the power margin of commercial-type small robots, the maximum power consumption of the five fingers was set to 15 W or less.

We designed and fabricated an SMA robot finger first followed by a five-fingered SMA robot hand, and evaluated whether the required specifications were achieved by two experiments. The joint angle and response time were measured using a single finger. As a result, by adjusting the duty ratio by PWM, the bending and extending times were 1.87 s and 1.63 s, respectively, and the joint angle reached 91% of the required specification. The power consumption was measured, while bending five fingers simultaneously using the five-fingered robot hand. It was observed that the maximum power consumption was 11.7 W. In conclusion, our small robot hand could satisfy all four requirements.

A questionnaire-based survey was also conducted with a robot hand. The typical gestures that are classified as symbolic gestures such as a peace sign and an OK sign were perceived well by participants. Dynamic gesture repeating the bending and extending such as “Come on” was difficult to perceive by participants. Many participants find it unnatural when multiple finger are out of sync. Not only response of individual finger, but driving of multiple fingers also needs to be evaluated. In the future, we also need to evaluate the characteristics of the gestures and the visibility from the user’s point of view. We further recommend the modeling of the torque generated around each joint by BMF and the bias spring in the future.

## Supplementary Information

Below is the link to the electronic supplementary material.

Supplementary material 1 (mov 54114 KB)

Supplementary material 2 (mov 53795 KB)

Supplementary material 3 (mov 56588 KB)
